# Genetic variant of *TTLL11* gene and subsequent ciliary defects are associated with idiopathic scoliosis in a 5-generation UK family

**DOI:** 10.1038/s41598-021-90155-0

**Published:** 2021-05-26

**Authors:** Hélène Mathieu, Shunmoogum A. Patten, Jose Antonio Aragon-Martin, Louise Ocaka, Michael Simpson, Anne Child, Florina Moldovan

**Affiliations:** 1 CHU Sainte-Justine Research Center, 3175 Côte Sainte-Catherine, 2.17.026, Montreal, QC H3T 1C5 Canada; 2 INRS-Centre Armand-Frappier Santé et Biotechnologie, Laval, QC H7V1B7 Canada; 3 Centre for Translational Omics–GOSgene, Department of Genetics and Genomic Medicine, UCL GOSH Institute of Child Health, 30 Guilford Street, London, WC1N 1EH UK; 4 NHLI, Imperial College, Guy Scadding Building, London, SW3 6LY UK; 5 Marfan Trust, NHLI, Imperial College, Guy Scadding Building, London, SW3 6LY UK; 6 Genetics and Molecular Medicine, King’s College London, SE1 1UL London, UK; 7 Faculty of Dentistry, Université de Montréal, Montreal, QC H3T 1J4 Canada

**Keywords:** Genetics, Gene expression, Genetic linkage study

## Abstract

Idiopathic scoliosis (IS) is a complex 3D deformation of the spine with a strong genetic component, most commonly found in adolescent girls. Adolescent idiopathic scoliosis (AIS) affects around 3% of the general population. In a 5-generation UK family, linkage analysis identified the locus *9q31.2-q34.2* as a candidate region for AIS; however, the causative gene remained unidentified. Here, using exome sequencing we identified a rare insertion c.1569_1570insTT in the tubulin tyrosine ligase like gene, member 11 (*TTLL11*) within that locus, as the IS causative gene in this British family. Two other *TTLL11* mutations were also identified in two additional AIS cases in the same cohort. Analyses of primary cells of individuals carrying the c.1569_1570insTT (NM_194252) mutation reveal a defect at the primary cilia level, which is less present, smaller and less polyglutamylated compared to control. Further, in a zebrafish, the knock down of *ttll11*, and the mutated *ttll11* confirmed its role in spine development and ciliary function in the fish retina. These findings provide evidence that mutations in *TTLL11*, a ciliary gene, contribute to the pathogenesis of IS.

## Introduction

Idiopathic scoliosis is a form of vertebral column deformity, defined as a combination of a deviation of the spine in the sagittal and coronal plane with a vertebral rotation. It is characterised by a Cobb angle of ≥ 10° curvature^1^ with rotation of the spine, both of which can be seen on an upright spinal radiograph^2^. A sub-type of scoliosis that mostly occurs in the adolescent years (between 8 and 18) during puberty, specifically during the skeletal growth spurt period, is called Adolescent Idiopathic Scoliosis (AIS). This sub-type has an overall prevalence of 0.47–5.2%, according to literature^3^. Affecting 3% of the population^4^, AIS is the most common spinal deformity and has been shown to affect females more than males with a ratio ranging from 1.5:1 to 3:1, increasing with Cobb angle measurements, from 1.4:1 in curves between 10° to 20° and up to 7.2:1 in curves > 40°^3^.

AIS is a multifactorial disorder and it is widely recognised that there is a genetic predisposition^5,6^. The mode of inheritance is still unclear, but several modes have been described^2,7^: autosomal dominant inheritance with partial penetrance^8^, X-linked inheritance^9^, sex-influenced autosomal dominant inheritance with a female:male ratio of 8:1^10^, and a complex trait or multifactorial mode of inheritance^11^. Indeed, 40% of AIS patients have a family history^12,13^, and there is a strong concordance between monozygotic twins compared to dizygotic twins^14^.

AIS is characterised by an important genetic heterogeneity^4^. Genetic analyses have identified several candidate loci predisposing to AIS. Significant evidence of linkage to various regions on chromosomes 6, 9, 16 and 17^15^ and the previously described locus on chromosome *19p13*^16^ has been independently confirmed. More recently, refinement of a major locus for AIS on chromosome *9q31.2-q34.2* and a novel locus on chromosome *17q25.3-qtel* were described^1,17^. All these studies reveal genetic heterogeneity and suggest polygenic aetiology of AIS^2^.

Due to genetic heterogeneity and phenotypic complexity, the aetiology of AIS remains unclear. Interestingly, several studies suggested the contribution of a specific organelle, the cilium^1,18–21^, in the pathogenesis of AIS. Human cilia are divided into two groups: motile and non-motile cilia (also called primary cilia), and their dysfunction leads to motor or sensory ciliopathies respectively^22^. It is well known that ciliopathies are associated with a high phenotypical heterogeneity including skeletal deformity (scoliosis)^23^. Primary cilia are chemical and mechanical sensors of pericellular environment thanks to different pathways like Wnt signaling–planar cell polarity pathway, signaling at focal adhesions and at adherents junctions, hedgehog signaling, notch signaling, and the JAK–STAT signaling^24^. Primary cilia have, among others, a role in mechanotransduction of fluid flow maintaining bone homeostasis especially in growth plate^25,26^ and in left-right patterning during embryogenesis^27^ which is impaired in AIS patients. Moreover, the identification of *POC5* [MIM: 617880], a ciliary gene, as an IS causing gene^1^, supports this hypothesis.

In this study, we identified a new candidate gene for IS. The susceptibility locus *9q31.2-q34.2* was first refined in a large UK family affected by IS presenting mainly in the adolescent period, and a mutation in the *TTLL11 * gene (Tubulin Tyrosine Ligase Like, member 11) [RefSeq: NM_001139442] was identified in a proband diagnosed with AIS. This gene has an important role in primary cilia integrity, and we showed that mutated TTLL11 lead to impaired ciliary glutamylation that resulted in shorter primary cilia in vitro using AIS patient’s fibroblasts. Moreover, we observed spinal deformity in vivo, using CRISPR-Cas9 zebrafish model, in both larvae and adult animals.

## Results

### Identification of a rare variant in *TTLL11* gene

**Figure 1:**
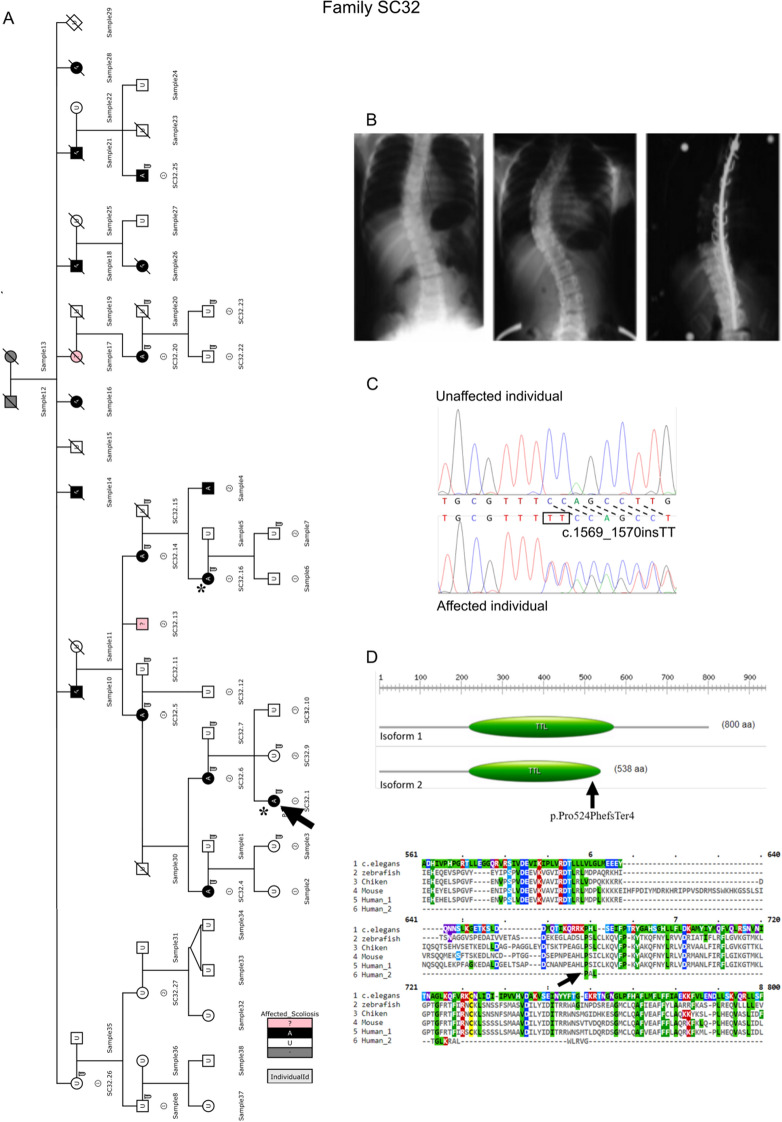
Pedigree of UK Family SC32 in which a putative gene mutation has been found. (**A**) Open circles and squares indicate unaffected individuals. Blackened circles and squares indicate affected females and males respectively. The arrow indicates family proband (SC32.1) and NGS sequenced patients are indicate by asterisks (SC32.1, SC32.16). (**B**) Standing posterior radiographs of proband V:3 of family SC32, showing right thoracolumbar spinal curvature in the absence of congenital vertebral abnormality. Cobb angle measurement of 40° at time of diagnosis (left panel), Cobb angle measurement of 56° before corrective surgery (middle panel), and spinal fusion (right panel). (**C**) Sequence obtained by Sanger sequencing of gDNA from scoliotic patient revealing the insertion of TT inducing a framshift (DE0193 lower chromatogram), compare to normal sequence of non-scoliotic patient (DE0194 upper chromatogram). (**D**) Representation of Human TTLL11 isoform 1 and 2 with the TTL domain showing the p.Pro524PhefsTer4 mutation (upper panel, black arrow), and partial alignment of TTLL11 by MUSCLE^96^ from different species to identify conserved domains (lower panel, black arrow represents the mutated proline).

Linkage analysis previously refined the region *9q31.2-q34.2* as an AIS susceptibility locus in a 5-generation family, SC32, affected by autosomal dominant AIS^2^ (Fig. 1A). The curvature in this family ranged from 15° to 65°, and the Cobb angle measured for the proband (SC32.1) was 40° at the diagnosis and progressed to 56° before surgery (Fig. 1B). The linkage analysis revealed that all clinically affected individuals within family SC32 shared a common haplotype, that was absent for all unaffected individuals, confirming that disease segregated with 9q (supplemental Figure 1)^2^. To identify the causative gene, exome sequencing was performed for this specific region, *9q31.2-q34.2*, on 2 affected patients, the proband SC32.1 and SC32.16 (3 meiosis distance, SC32 family). Each exome was sequenced to a mean depth of 71.39× and 62.87× with > 84.01% and > 81.18% of coding bases covered by > 20 reads respectively.

Reads were then aligned to the reference genome hg19 and a rare variant in *TTLL11* gene was identified by variant calling (Fig. 1C). *TLL11* gene codes for two transcripts, transcript 1 (NM_001139442), which is longer than transcript 2 (NM_194252). The identified mutation is an insertion of two thymine (g.124751443_124751444insTT, MAF < 1%) that results in a premature stop codon in the transcript 2 (c.1569_1570insTT), leading to a truncated protein (p.Pro524PhefsTer4) (Fig. 1D). This rare variant that we identified was not present in 3000 in house control exomes and was found on 8 alleles on 8246 according to the EVS database (Exome variant server^28^) (supplemental Table 1). Moreover, this variant is predicted to be pathogenic for the transcript 2 by UMD-predictor^29^ (UMD-score: 100) and MutationTaster^30^ (score: 1; disease causing).

Subsequently, to assess for mutations in *TTLL11* in a larger cohort, its 9 exons were screened by Ion AmpliSeq from Life technology in 96 AIS affected individuals from a French-Canadian and British population. The VarAFT 2.3 software was used to annotate and filter the 13 genetic variants discovered in *TTLL11* gene. After filtering (minor allele frequency < 1%, single-nucleotide substitutions and small indels, pathogenicity prediction), out of 13 variants, 2 were identified as novel rare variants in *TTLL11 * (Supplemental Table 2). Interestingly, these two rare variants were found in 2 British AIS patients and none were identified in any of the AIS affected patients from the French-Canadian population (Supplemental Figure 2). These two mutations are predicted to be pathogenic for the transcript 1 (NM_001139442), SC203: c.1751+1G>A, (rs not known), HSF: Broken WT Donor Site and SC217: c.2214C>A; rs766983167, UMD-score: 100).

**Figure 2:**
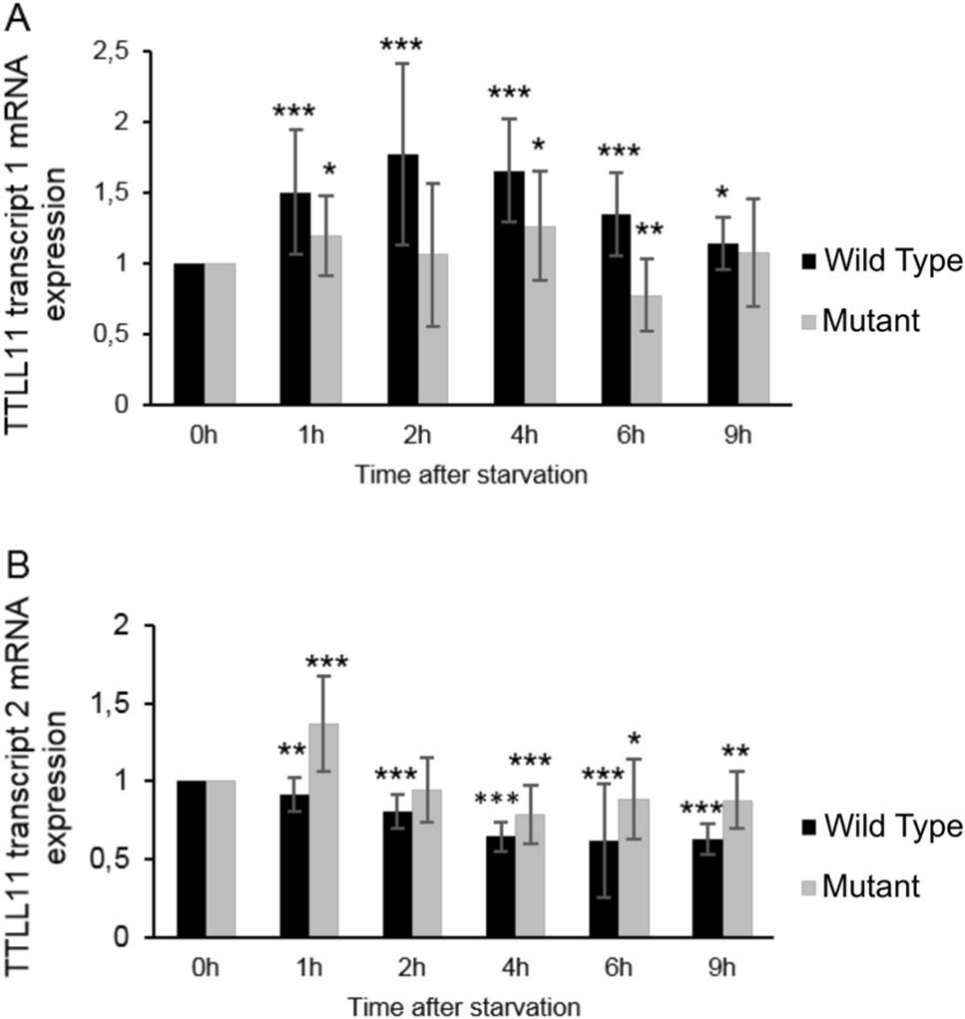
WT and mutant TTLL11 mRNA expression after starvation. WT (DE0194) and mutant (DE0193) fibroblasts were treated with medium without FBS for different times. Cells were then processed for mRNA isolation and the relative mRNA expression of both transcript 1 (**A**) and 2 (**B**) of TTLL11 at different time point after serum starvation were determined by qPCR. The levels of mRNAs were plotted relative to cells harvested at 0 h (n = 3). Error bars represent SD. The difference between one independent group and the control group 0 h was examined by unpaired, two-tailed Student’s t-test, *P ≤ 0.05, **P ≤ 0.01, ***P ≤ 0.001.

To gain more insights on the pathogenic role of the mutation in gene *TTLL11 *on its expression, we first assessed *TTLL11* transcripts 1 and 2 expression from healthy control (DE0194) and patient-derived fibroblasts (NM_194252: c.1569_1570insTT; DE0193). In WT cells, the expression of WT *TTLL11* transcript 1 was increased from 1 to 2 h following FBS privation prior to returning to regular expression (Fig. 2A). In parallel, the mRNA expression of WT *TTLL11* transcript 2 was decreased (Fig. 2B). In mutant cells, the expression of *TTLL11* transcript 1 is reduced compared to WT transcript (Fig. 2A). In parallel, mutant *TTLL11* transcript 2 expression is increased compared to WT (Fig. 2B). These results suggest that the c.1569_1570insTT mutation disturbs the mRNA expression of *TTLL11 *transcript 1 and 2.

### TTLL11 localisation and ciliogenesis

**Figure 3:**
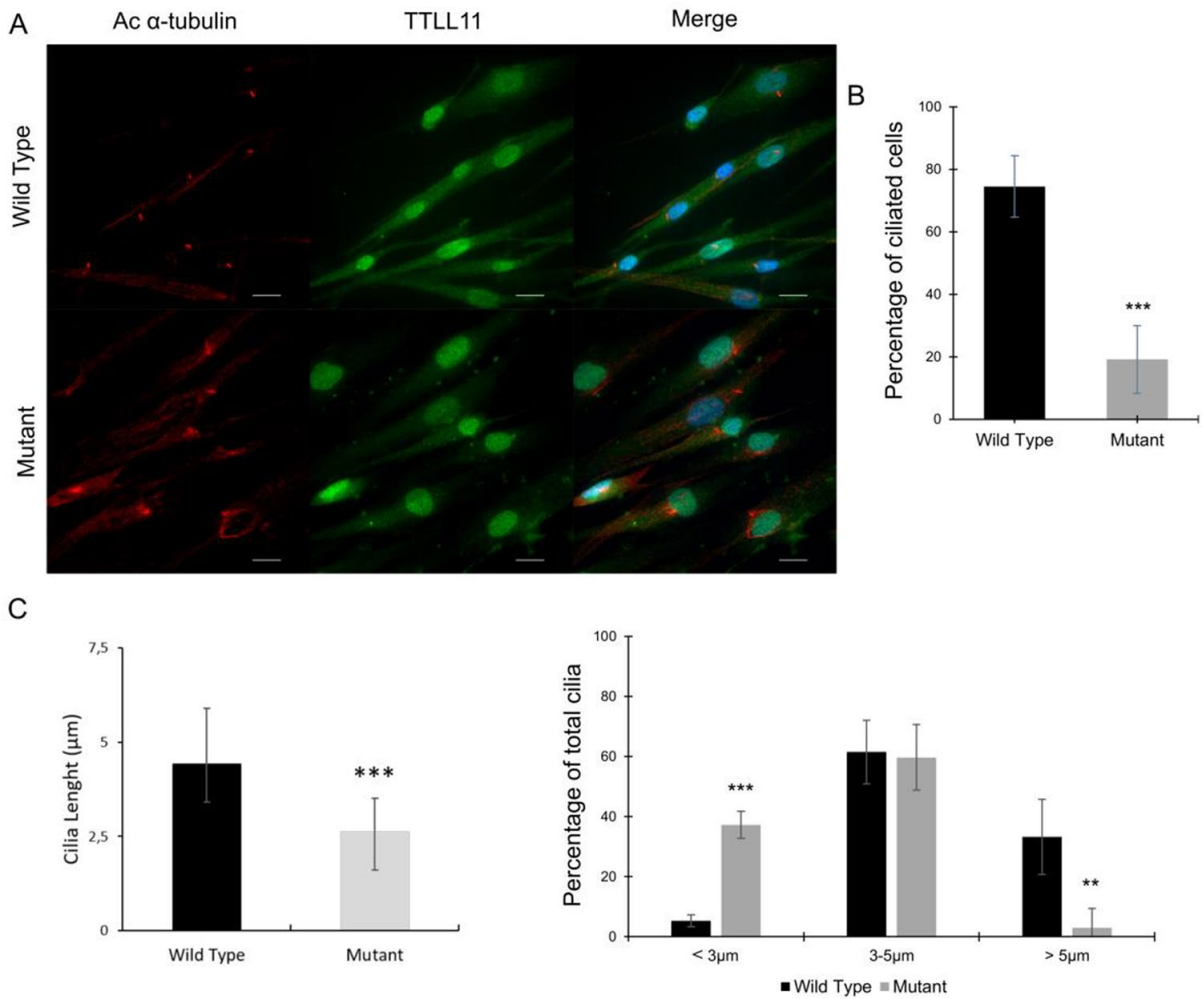
WT and mutant TTLL11 protein expression after starvation. (**A**) WT (DE0194) and mutant (DE0193) fibroblasts were treated with medium without FBS for 24 h. Cells were stained with immunofluorescence for acetylated $\upalpha$-Tubulin (red), TTLL11 (green) and DAPI (blue). Scale bar 20 μm. (**B**) Graphical representation of the percentage of TTLL11 mutant ciliated fibroblasts compared to WT fibroblasts (DE0194) n = 660. (**C**) Graphical representation of mean cilia length for TTLL11 mutant ciliated fibroblasts compared to WT fibroblasts (left panel) and distribution of ciliated cells based on cilia length: < 3 $\upmu$m; 3–5 $\upmu$m; > 5 $\upmu$m (right panel); n = 185. Values shown as mean ± SD, the difference with controls was examined by unpaired, two-tailed Student’s t-test, *P ≤ 0.05, **P ≤ 0.01, ***P ≤ 0.001.

The localization of TTLL11 was assessed by immunofluorescence staining in mutant DE0193 and WT DE0194 fibroblasts after 24 h of serum starvation^31,32^. Acetylated-$\upalpha$-tubulin antibody binds to $\upalpha$-tubulin that carry acetylated K40, meaning primary cilia from proximal to distal end^33^, centrioles, mitotic spindles, midbodies and to subsets of cytoplasmic microtubules. According to these results and assuming that $\upalpha$-tubulin acetylation of primary cilia is independent of TTLL1 function and reliably indicates ciliary length, acetylated-$\upalpha$-tubulin was used as primary cilia marker. Despite the ciliary function, in fibroblasts, WT TTLL11 proteins localized in the nucleus and in the cytoplasm (Fig. 3A) but mutant TTLL11 proteins were more nuclear.

Moreover, after 24 h of serum starvation, around 75% of WT fibroblasts showed acetylated $\upalpha$-tubulin primary cilia compared to 20% for the TTLL11 mutant fibroblasts (p = 3.9 × 10${}^{-9}$) (Fig. 3B) for which the length of acetylated $\upalpha$-tubulin from the primary cilium was reduced (Fig. 3C). Indeed, primary cilia of more than 5 μm are significantly reduced for mutant cells, and cilia of less than 3 μm were more prominent in mutant cells compared to WT fibroblasts (Fig. 3C). These results reveal that the identified mutation (c.1569_1570insTT) of *TTLL11* leads to a mislocalisation and shorter cilia or the absence of cilia. Taken together, these results confirm the implication of TTLL11 in primary cilia integrity.

### *TTLL11 *is required for proper long glutamate side chains

**Figure 4:**
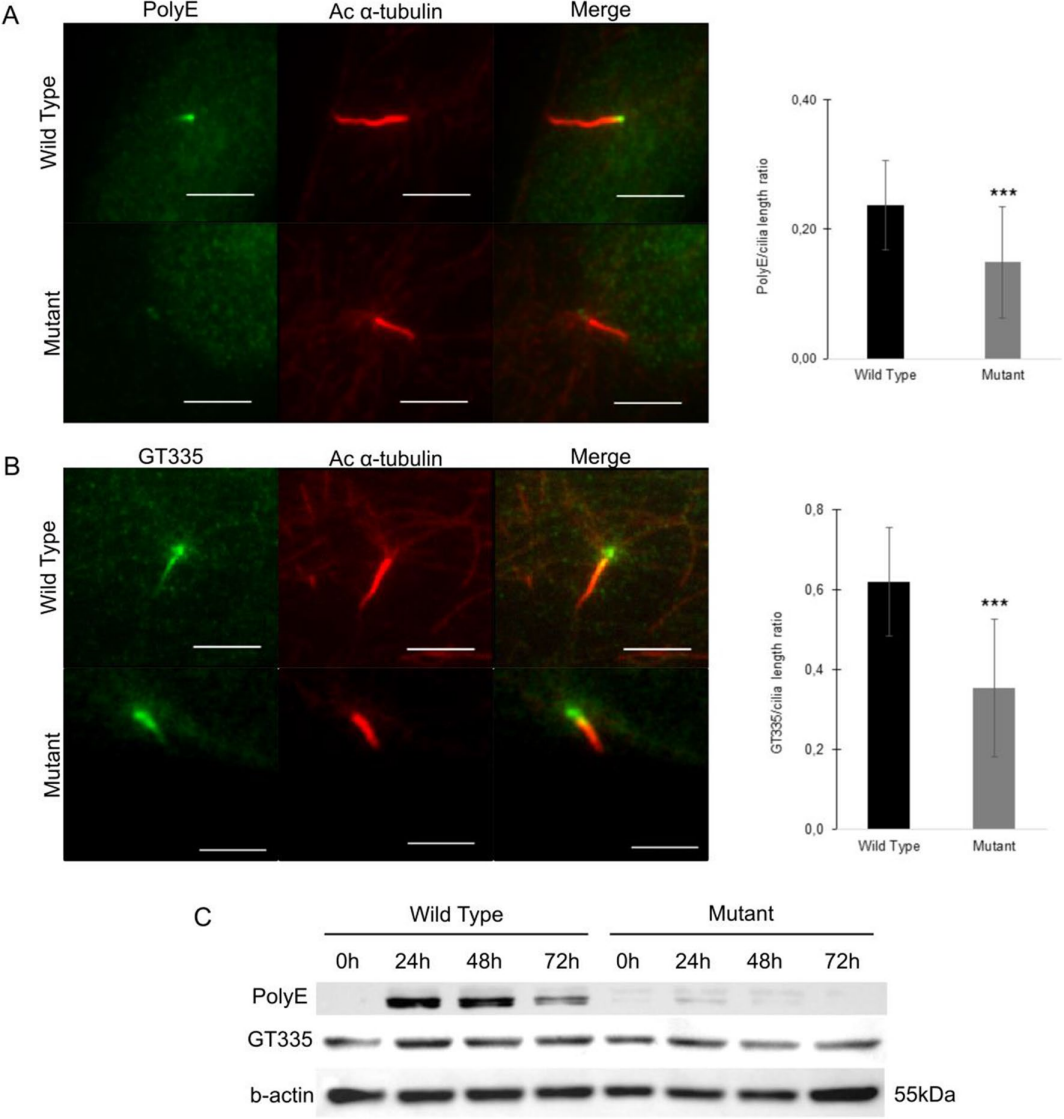
Cilia elongation requires long polyglutamate chain generated by TTLL11. (**A**) WT (DE0194) and mutant (DE0193) fibroblasts were treated with medium without FBS for 24 h. Left panel, cells stained with immunofluorescence for acetylated $\upalpha$-Tubulin (red) and PolyE (green) show a strong reduction of polyglutamylation for the mutant TTLL11 cells. Scale bar 5 $\upmu$m. Right panel, Quantification of the PolyE/cilia length ratio in mutant ciliated fibroblasts (DE0193) compare to WT ciliated fibroblasts (DE0194). n = 75. (**B**) Wild type (DE0194) and mutant (DE0193) fibroblasts were treated with medium without FBS for 24 h. Left panel, cells stained with immunofluorescence for acetylated $\upalpha$-Tubulin (red), GT335 (green) and DAPI (blue) show a decrease of polyglutamylation for the mutant TTLL11 cells. Scale bar 5 $\upmu$m. Right panel, Quantification of the GT335/cilia length ratio in mutant ciliated fibroblasts (DE0193) compare to WT fibroblasts (DE0194). n = 118. (**C**) Protein polyglutamylation of Wild type (DE0194) and mutant (DE0193) fibroblasts treated with medium without FBS for 0, 24, 48 and 72 h analysed by western blot (GT335 and PolyE antibodies) reveal the loss of long polyglutamylate chains for the mutant DE0193 cells. Full gels are shown as supplementary data. Values shown as mean ± SD, the difference between two independent groups was examined by unpaired, two-tailed Student’s t-test, ***P ≤ 0.001.

Tubulin undergoes different post-translational modifications (PTMs) called the tubulin code. The most characterised PTMs are acetylation, detyronisation, glycylation and glutamylation^34^. TTLL11 is a polyglutamylase from the tubulin tyrosine ligase-like (TTLL) family of proteins that includes enzymes responsible for the two PTMs glutamylation and glycylation. TTLL11 is known to preferentially modify $\upalpha$-tubulin by extending the glutamate chain as an elongase^35^. The functionality of TTLL11 in mutant DE0193 cells and WT DE0194 cells was then assessed using both GT335 and polyE antibodies that detects the branch point of glutamate side chains or the long polyglutamate chains (≥ 3 glutamates) respectively. Interestingly, the polyglutamate chains were reduced at the cilium level in mutant cells (Fig. 4A,B), and the amount of branch point glutamate was also reduced (Stained with GT335). Additionally, the long polyglutamate chains (≥ 3 glutamates) were induced after serum starvation until 48 h prior to decrease in the WT cells while the long polyglutamate chains were strongly decreased in the mutant TTLL11 cells (Fig. 4C); leading to short cilium length. These results demonstrate that TTLL11 is required for proper long glutamate side chains elongation, and this function is impaired in *TTLL11* mutant AIS cells.

### *Ttll11 *is implicated in zebrafish spinal development

**Figure 5:**
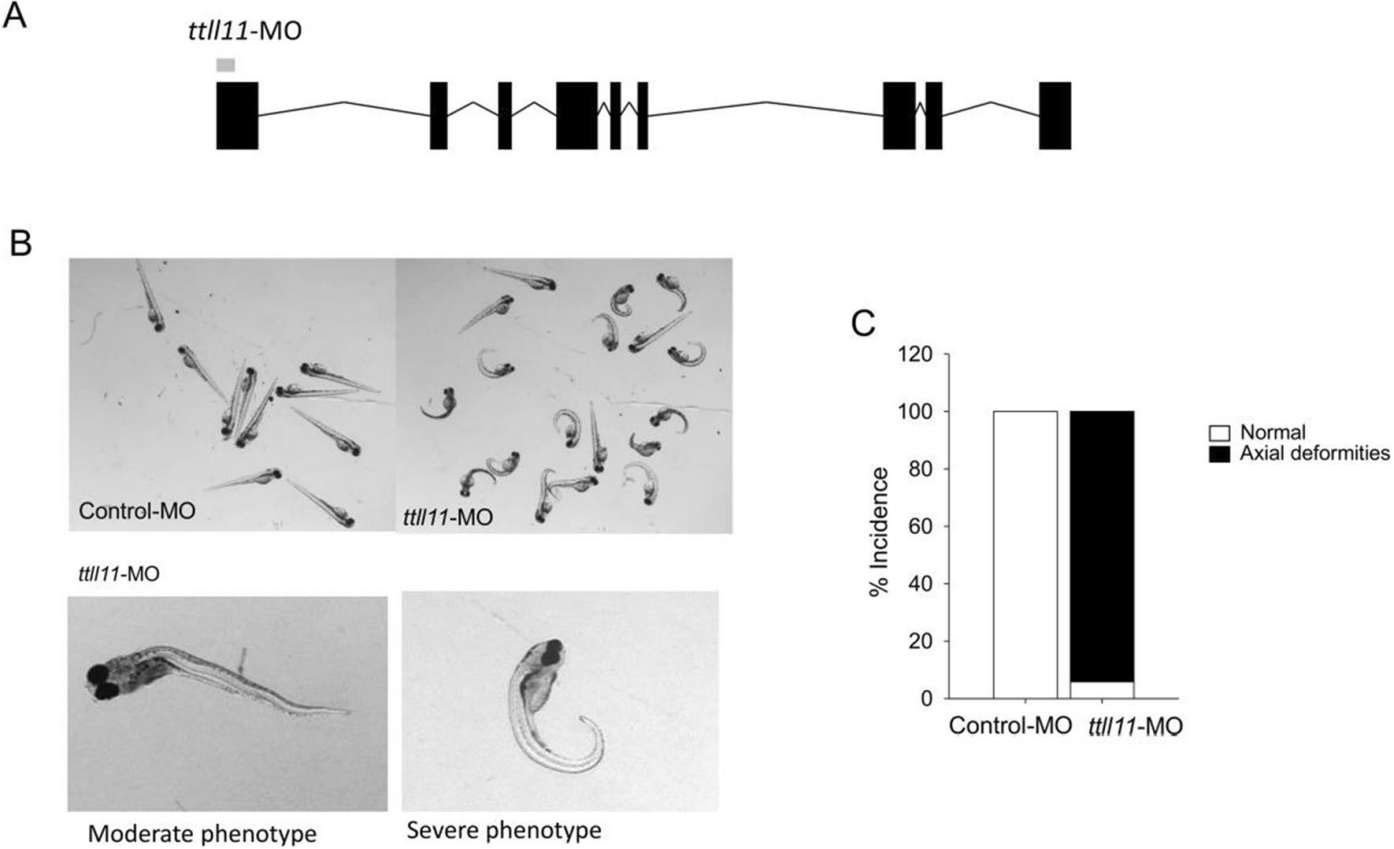
Knockdown of *ttll11* leads to scoliosis phenotype in zebrafish larvae. *Ttll11* knock-down zebrafish showed body curvature phenotype compared to control. (**A**) Representation of the blocking antisense morpholino oligonucleotide targeting zebrafish *ttll11* (NM_001077375.1). (**B**) Mutant and control zebrafish phenotype (3–5 dpf). (**C**) Incidence of body axial deformity after MOs injection into fertilised zebrafish eggs at the one- to two-cell stages.

To further functionally validate the implication of *TTLL11* in the pathogenesis of AIS, knockdown modelling in zebrafish was performed; a powerful model to study AIS^1,18,36,37^. Phylogenetic analysis indicated that *TTLL11* was conserved during evolution and that zebrafish have a unique ortholog that shares 71% of homology with human *TTLL11* gene. Interestingly, *ttll11* is expressed as early as the 8-cell stage and later in development its expression is predominantly localized to the central nervous system^38^. We disrupted *ttll11* expression using an antisense morpholino (MO) (Fig. 5A). Loss of function of *ttll11* resulted in a significant curvature of the body axis in ttll11 morphants compared to control embryos as from 2-days postfertilization (hpf) (Fig. 5B). The body curvature in ttll11 morphants progresses through early larval stages (4–6 dpf) with a moderate or severe body curvature (Fig. 5C). Similar phenotypic variability in the appearance and severity of the curve has been observed in several zebrafish models of scoliosis^1,39,40^. We were unfortunately not able to fully characterize the spinal curvature during juvenile and adult stages using bone imaging techniques because the ttll11 morphants did not survive past 9–12 dpf, likely due to their inability to swim and reach for food.

**Figure 6:**
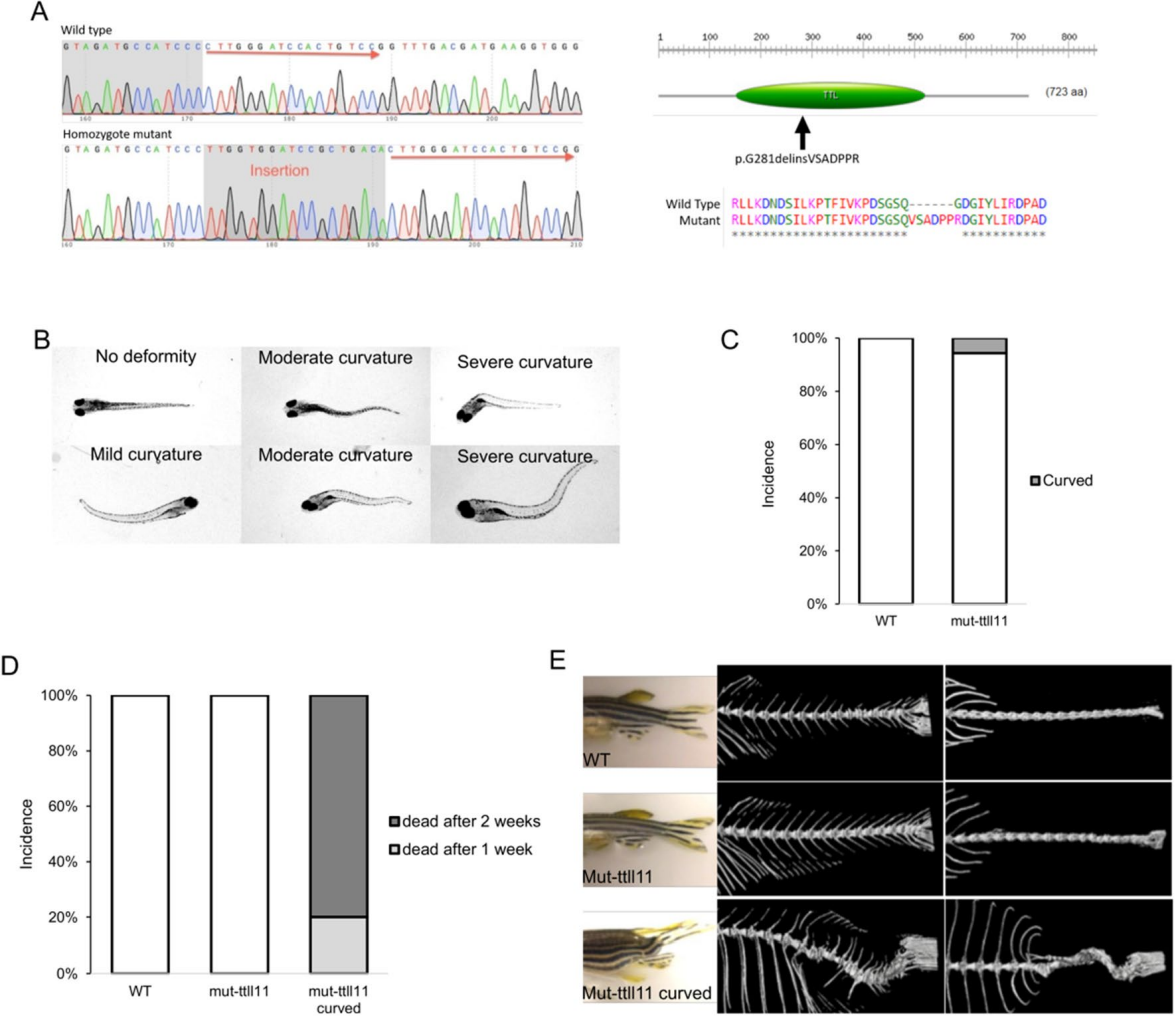
Effect of *ttll11*-mut in zebrafish body axis and lethality. (**A**) Sequence obtain by Sanger sequencing of the mut-*ttll11* zebrafish (left panel, lower chromatogram) compared to WT (left panel, upper chromatogram) and protein consequences (right panel). (**B**) Mut-*ttll11* display different type of 3D curvature of the body axis in 8 days old mut-*ttll11* zebrafish. (**C**) Incidence of scoliosis phenotype in 8 days old mut-*ttll11* zebrafish. 6% of the mut-*ttll11* zebrafish show a 3D curvature of the body axis. n = 250. (**D**) Lethality of scoliosis phenotype in 8 days old mut-*ttll11* zebrafish. All the mut-*ttll11* zebrafish expressing 3D curvature died after 2 weeks, 20% the first week and 80% the second week. All the WT and mut-ttll11 no curved were alive after 2 weeks. n = 15. (**E**) lateral view (left panels) of WT, mut-*ttll11* and mut-*ttll11* with scoliosis adult zebrafish. MicroCT lateral (middle panels) and dorsal (right panels) images reveal a 3D curvature for the mut-*ttll11* with scoliosis fish compared to WT and mut-*ttll11* without scoliosis phenotype.

We thus created a mutant zebrafish line for *ttll11* gene using CRISPR-Cas9. We identified a founder mutant zebrafish carrying an 18 bp insertion in exon 4 of *ttll11 *leading to a strong modification of the TTL domain of the protein (Fig. 6A). The zebrafish homozygous mutant (mut-*ttll11*) exhibited a high incidence of body axis curvature in the larvae compared to WT fish but at a lower penetrance than *ttll11*-MO (Fig. 6C). The body curvature varied with different degrees of severity (Fig. 6B). Moreover, all the mutant larvae that were showing a curly phenotype died before 2 weeks post fertilization (Fig. 6D). Fish that acquired the curvature at a later stage were able to live until adult stage and we analysed mineralized spine of these adult fish using micro-CT that revealed a 3D deformation similar to the deformation observed in AIS patients (Fig. 6E and supplemental videos). Indeed, the mutant spine displays curvature in two different planes with a rotation of vertebrae.

### *Ttll11 * is implicated in retinal integrity in zebrafish

**Figure 7:**
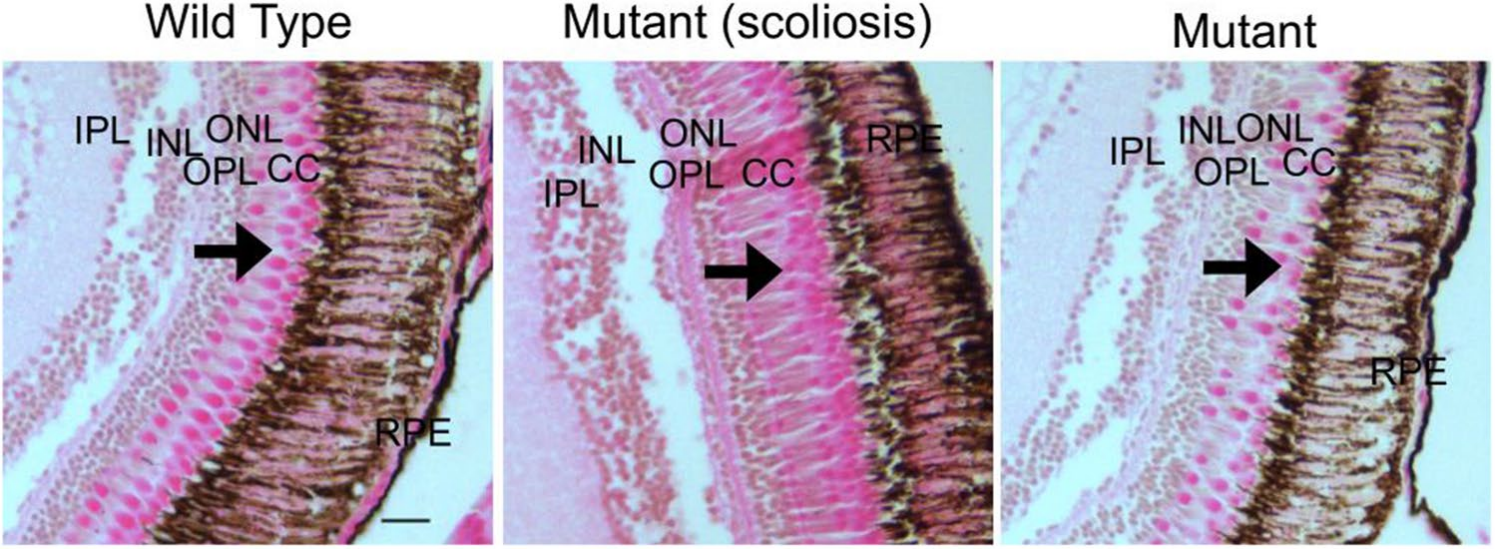
Hematoxylin and eosin (H&E)-stained retina of WT and mutant *ttll11* adult zebrafish. Retinal histology reveals a disorganization of the cone cell layer from *ttll11* mutant adult zebrafish compared to the WT (black arrows). The structure and the number of cone cells are impaired. n = 3. Scale bar 20 $\upmu$m. RPE, retinal pigmented epithelium; CC, Cone cell layer; ONL, outer nuclear layer; OPL, outer plexiform layer; INL, inner nuclear layer; IPL, inner plexiform layer; GCL, ganglion cell layer.

Ciliary defects cause a group of diseases called ciliopathies that are especially characterized by retinal defects and scoliosis. Cilia, as a complex structure, comprises more than 900 genes that are involved in ciliary structure and function^41^, and those genes are called ciliary genes. Because* TTLL11* is a ciliary gene, and ciliary genes, such as *POC5,* were already related to retinal function^42–45^, the ciliary retinal tissue was then investigated. Zebrafish *ttll11* has been reported to be expressed within the CNS and neural tube^38^. Moreover, function and structure of photoreceptor cilia are well understood^46^ and zebrafish retina is easy to access. Retinal sections of WT and mut-*ttll11* zebrafish with and without scoliosis were stained by hematoxylin eosin (Fig. 7) and revealed a disorganization of the cone cell layer of mut-*ttll11* adult zebrafish compared to control.

**Figure 8:**
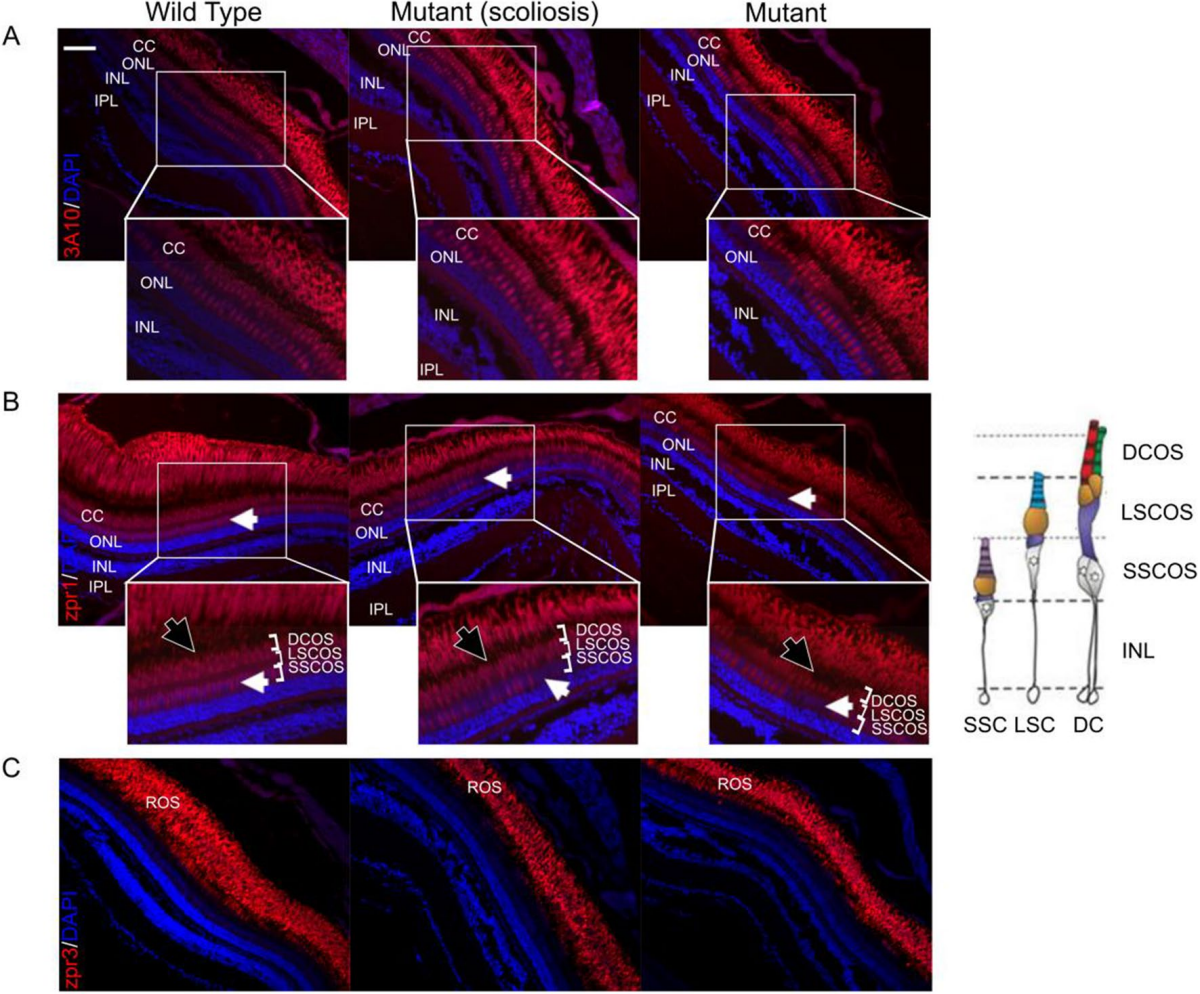
Effect of mutant *ttll11* on adult zebrafish rod and cone photoreceptors. (**A**) Retinal sections of WT and mut-*ttll11* zebrafish with and without scoliosis, labeled with 3A10 antibody (red) and DAPI (blue). 3A10 antibody is specific to neurofilaments (rods and cones). This labelling shows a disorganisation of the cone cells layer. Scale bar: 20 $\upmu$m. (**B**) Retinal sections of WT and mut-*ttll11* zebrafish with and without scoliosis, labeled with zpr1 antibody. The specific labelling of double cones with zpr1 antibody suggest a disorganisation of cones cell body layer (white arrows) and the layer of double cones cilium seems to be impaired (black arrows). Right panel, representation of cone cell layer organisation, adapted from Lagman et al.^97^. (**C**) Retinal sections of WT and mut-*ttll11* zebrafish with and without scoliosis, labeled with zpr3 antibody. Zpr3 labelling is specific to rods and doesn’t show significant difference between WT and mutant zebrafish. n = 3. ROS: Rod Outer Segment; CC: Cone cell layer; OS: Outer Segment; ONL: Outer Nuclear Layer; IPL: Inner Plexiform Layer; INL: Inner Nuclear Layer; GCL, ganglion cell layer; DCOS : double cone outer segment; LSCOS: long single cone outer segment; SSCOS: short single cone outer segment.

To validate this finding, retinal sections were then labeled with different retinal layer specific antibodies: 3A10 antibody, specific to neurofilaments (rods and cones), zpr1 antibody, specific to cones and zpr3 that is specific to rods (Fig. 8). We observed a disorganisation of the cone cell layer (Fig. 8A,B) and especially of the double cones in mutant fish compared to controls. Indeed, lack of staining is observed in cones cell body layer (Fig. 8B, white arrows) and the layer of double cones cilium seems to be impaired (Fig. 8B, black arrows). On the other hand, no anomalies were observed in the rod layer between mutant *ttll11* zebrafish and controls (Fig. 8C). These results support the implication of the ciliary protein ttll11 in retinal layer integrity in a zebrafish model.

## Discussion

Idiopathic Scoliosis is a complex disease with a multifactorial aetiology^47^ including genetic, epigenetic^48^, environmental, biomechanical^49^ and hormonal^50^ factors. Many studies identified candidate genes that are probably involved in pathogenesis of IS and AIS but almost all of them are susceptibility genes, not causative genes^51,52^. In this study, we reported a new causative gene in a five-generation UK family, *TTLL11,* a ciliary gene.

The refinement of a major locus for IS on chromosome 9q31.2-q34.2^2^,*TTLL11* disrupting the transcript 2 of this gene (NM_194252). Sequencing of *TTLL11*,*TTLL11*,*TTLL11*,

TTLL11 is a ciliary protein and a member of Tubulin Tyrosine Ligase Like (TTLL) family, which play a role in $\upalpha$- and $\upbeta$-tubulin mono- and polyglutamylation, a post-translational modification (PTM). This PTM is crucial for ciliary integrity, cell cycle and cell differentiation^53,54^. Indeed, polyglutamylation has been shown to maintain a proper alignment of outer doublet in ciliary axoneme^55,56^.

Recently Patten et al.^1^ reported a causative gene, *POC5,* a ciliary-related gene, which explains 10% of AIS family cases. *TTLL11* and *POC5* seem to be involved in a similar biological process, which could be involved in the physiopathogenesis of AIS. Indeed, these genes have an important role in the integrity of cilia especially primary cilia. It is accepted that ciliopathies could include skeletal deformity and scoliosis.

Polyglutamylation is a reversible modification characterized by the addition of glutamate residue, carefully regulated by the TTLL proteins family and tubulin deglutamylase of the Cytoplasmic Carboxyl Peptidase (CCP) proteins family^57^. This post-translational modification allows the interaction with the microtubule-associated proteins (MAPs)^54^ and play a role on intraflagellar transport (IFT)^58^.

In human fibroblasts, we showed that TTLL11 is localized in the nucleus. Similar findings were observed when TTLL11 was expressed in HeLa cells^59^. In contrast, ttll11 was shown to localize in the cilium in *C. elegans*^60^ and in the basal body in MDCK cells^35^. However, these are limited studies and further work is required to better characterize its cellular localization in mammalian cells, especially in human cells.

According to the literature, mouse Ttll11 shows preference for polyglutamylation elongation of $\upalpha$-tubulin when overexpressed in HeLa cells^35^, but in the nematode *C. elegans*, a recent study highlighted the crucial role of *ttll11* to initiate the glutamate side chain^61^. This recent analysis is consistent with our data in human AIS cells, indeed we showed that TTLL11 was required to add the initiating glutamate to the side chain and proper long glutamate side chain. We noticed that the GT335 signal was not completely lost suggesting that some residual branch point glutamates were still added, suggesting a residual activity of mutant TTLL11, or a residual activity of one of the other initiase from the TTLLs family. For instance, TTLL5 is responsible of the proper glutamylation of RPGR, an X-linked retinitis pigmentosa GTPase regulator^62^. To date, no non-tubulin substrate for TTLL11 has been described, but it can’t be excluded.

Little is known about the role of TTLL11 and about its potential role in IS but recent studies demonstrate that cilia hypoglutamylation promotes cilia disassembly^63^ and affect Hh signalling through anterograde IFT-dependant mechanism^58,63^. *TTLL11* gene lead to two different transcripts, transcript 1 composed by 9 exons and transcript 2 that has 4 exons, but their roles are not well documented yet.

Our experiments support the hypothesis of the implication of cilia pathway in AIS physiopathogenesis. Moreover, the TTLL protein family was found to be involved in Joubert syndrome [MIM; 612291], a ciliopathy characterized by mid-hindbrain malformation, hypotonia, developmental delay and skeletal defects such as scoliosis^64^. Indeed, TTLL6, which is also an $\upalpha$-tubulin polyglutamylase^53^, was found to be mislocalized in Joubert Syndrome^65^. The characterization of TTLL11 mRNA expression and its localization in AIS patient fibroblasts highlight an impairment due to the c.1569_1570insTT mutation and this seems to drastically affect ciliogenesis. An increase of transcript 2 was observed, and although in-silico prediction, we also observed a modification of TTLL11 transcript 1 mRNA expression, probably to balance the over-expression of transcript 2. Altogether, our results suggest that the altered TTLL11 protein leads to a hypoglutamylated tubulin resulting in shorter primary cilia. Supporting this hypothesis several studies have shown the involvement of glutamylation in ciliogenesis, IFT and Hedgehog signaling^58,63^. Moreover, TTLL11 glutamylation was demonstrated to regulate ciliary trafficking, extracellular vesicle (EVs) release in *C. elegans*^60^.

Interestingly, cilia host Ihh signalling^66^, which is involved in both endochondral and intramembranous ossification^67^, that are implicated in spine development^68^. In addition, Ihh pathway is associated with height^69,70^, an important parameter for the evolution of the spine curvature occurring in AIS^71^. Primary cilia host also the polycystin pathway which is involved in skeletogenesis and bone development^72,73^. Moreover, cilia sensory functions are exhibited by the TRP family receptors and Ca^2+^ influxes and interestingly, cilia respond to the sex hormones^74,75^ that are significantly increased during puberty.

To support the implication of this gene in IS physiopathogenesis we did functional studies in an animal model, zebrafish (*Danio rerio*). The phenotype that we observed is similar to the phenotype observed in fish when *ttll3* and *ttll6*, two other members of the TTLL protein family, are knocked down^38^, which supports the implication of this family in skeletal development. Because this approach is not highly specific and because these larvae did not live for more than 2 weeks, a mutant zebrafish line was designed using the genome-editing technology CRISPR-Cas9. With the selected guide, only one mutation was obtained, an 18pb insertion in exon 4 of *ttll11 * leading to an insertion of 6 amino acids on the TTL domain of the protein. Almost no larvae with spine curvature were able to stay alive suggesting that this deformation is not viable for more than 2 weeks. This effect could be explained by the fact that the curvature affects their swimming capacity and prevents them from feeding. However, some fish developed this deformity at a later stage and were able to grow until adult stage. The curvature observed by micro-CT analysis in this *ttll11* mutant zebrafish line is a 3D spine curvature with vertebral rotation (supplemental videos).

Because TTLL11 is a ciliary protein and because the TTLL5 (also from the TTLLs family) and the ciliary protein POC5 was found to be implicated in scoliosis and retinal function^1,42,62^, histology of mut-*ttll11* retinal tissue was assessed and revealed a disorganisation of the double cone cell layer. Interestingly, no retinal defect was initially observed in *Ttll5*${}^{-/-}{}^{\ }$ mice^76^, but further additional assays at older stage revealed a progressive photoreceptor degeneration^62^. These studies describe that retinal defect was seen in older animals but AIS is a disease that develop during the puberty, at younger stage of life.

These results suggested a link between vision and the proper development of the spine as a link between vision and AIS was already showed in human^77^, this field warrants further investigations. Noteworthy, no visual disturbance was observed in the 5-generation UK family based on clinical record.

In parallel, several studies showed that cilia could be involved in AIS by their implication in CSF (cerebrospinal fluid). Indeed, motile cilia of the surrounding epithelium of CSF could play an important role in distribution of this flow^78^, and a defect of the CSF flow in zebrafish influences body axis formation and spine morphogenesis^18,79,80^ possibly through CSF-contacting neurons that line the central canal of the spinal cord and the brain ventricles^80^.

Finally, identifying *TTLL11*, as a novel gene for IS with role in cilia biology is a crucial step to clarify pathways potentially involved in this condition. Based on this study, in addition to the previously identified ciliary genes in Human^1,81–85^ and animal model^19,37,39^, this pathology could be considered as a cilia-associated disorder, or a subtype of ciliopathy.

Further studies of larger cohort are needed to establish the prevalence of mutated *TTLL11* in AIS and to clarify the involvement of ciliary pathways. Understanding these mechanisms can contribute, in the long term, to the early diagnosis of scoliosis and may help to establish strategies to prevent the progression of deformity. Meanwhile, in affected families were causative or predisposing mutations are identified, offspring can be screened in the new-born period. Those identified as carrying the family mutation can be followed at regular intervals and early spinal curvature treated optimally to prevent severe deformity.

## Material and methods

### Patients

A five-generation British family (SC32), with eighteen known affected members (clinical data published in^2^, supplemental Table 3), was recruited by the orthopaedic surgeon at St George’s Hospital, London, UK. The proband SC32.1 was diagnosed as having adolescent idiopathic scoliosis (AIS). Among the family members, 7 males and 11 females (Fig. 1A) were affected. In total, there are nine affected members living. All are affected by a lateral spine curvature of at least 10° with vertebral rotation (Fig. 1B) and the age of diagnosis was variable. Any recognised syndrome including Marfan syndrome was ruled out. This family’s mode of inheritance of disease is autosomal dominant with a strong pattern of adolescent idiopathic scoliosis. An additional collection of 96 patients composed by 63 unrelated British consecutives IS individuals and 10 families, and 18 French-Canadian families affected with autosomal dominant IS was established. The population control consisted of 3000 individuals not screened for IS.

### Whole exome sequencing

Whole exome sequencing was performed by the Division of Genetics and Molecular Medicine (King’s College London, UK) using DNA extracted from whole blood in two affected individuals from family SC32.1 and SC32.16. This strategy, where we assumed that rare or low-frequency variants shared between the two exomes sequenced affected relatives in a family, were highly likely to be identical, optimised the information gained and reduce the cost compared to WES of all the affected individuals. A total of 3 μg of genomic DNA was sheared to a mean fragment size of 150 bp [Covaris], and the fragments used for Illumina paired-end DNA library preparation and enrichment for target sequences [SureSelect Human All Exon 50Mb kit, Agilent]. Enriched DNA fragments were sequenced with 100 bp paired-end reads [HiSeq2000 platform, Illumina]. Sequencing reads were aligned to the reference human genome sequence hg19 using the Novoalign software^86^. Duplicate and multiply mapping reads were excluded, and the depth and breadth of sequence coverage were calculated using custom scripts and the BedTools package^87^. Single-nucleotide substitutions and small indels were identified with SAMtools^88^, annotated with the ANNOVAR software^89^. The identified SNPs were then compared to a 3000 controls cohort and EVS database^90^.

### Sanger sequencing

DNA was sequenced using ABI BigDye terminator cycle sequencing chemistry 3.1 kit (Applied Biosystems, Foster City, CA) on an ABI 3100 automated sequencer (Applied Biosystems) according to manufacturer’s instructions. Cycle sequencing of sequencing reactions was conducted on a PTC-100 Peltier Thermal Cycler (MJ Reseach). The sequencing reactions were purified using DyeEX 2.0 Spin Kit or DyeEx 96 plate (QIAGEN) according to manufacturer’s instructions (QIAGEN). The purified sequencing reaction was dried on a thermal cycler at 80 °C. The resultant DNA pellet was then resuspended in 12 μl of deionised formamide and loaded into a 96 well non-skirted PCR plate (ABGENE). Prior to loading onto the ABI 3100 genetic analyser for sequencing (Applied Biosystems), samples were denatured at 95 °C for 5 min and cooled on ice.

### *TTLL11-*exome sequencing

A library was designed to perform the targeted sequencing of the *TTLL11* gene using the Ion AmpliSeq (life technology). Sequencing of the 9 exons of *TTLL11* of 63 unrelated British consecutives AIS individuals and 10 families, and 18 French-Canadian families affected with autosomal dominant AIS was performed by Centre de Génomique Clinique Pédiatrique intégré CHU Sainte-Justine. The library was prepared using the Ion AmpliSeq DNA and RNA Library Preparation (MAN0006735, Rev. B.0, Ion Torrent, Life technologies) prior to the exome sequencing following the Ion PGM IC 200 Kit (MAN0007661, Rev. B.0) protocol. Sequencing reads were aligned to the reference human genome sequence [hg19] and the SNPs were identified by Ion Reporter [Ion Torrent]. Identified variants were annotated using ANNOVAR^89^ which is implemented in VarAFT software^91^ which we used to select exonic and splicing variants with a MAF (minor allele frequency) ≤ 1%.

### Validation with Sanger sequencing

Identified variants were validated by Sanger sequencing. PCR amplification was performed using the TransStart FastPfu FLY DNA Polymerase (AP231, civic bioscience) following instructions of the manufacturer with primers described in supplemental Table 4. Sanger sequencing of amplicons was performed on an ABI 3730xl DNA Analyzer (Applied Biosystems) at McGill University and Génome Québec Innovation Center (Montréal, Canada).

### Cell culture

Human Fibroblasts (DE0194 HB6574 and DE0193 HB2981) were provided by Dr. Anne H Child (Department of Cardiological Sciences, St George’s Medical School, University of London, UK). Cells were maintained in HAM’S F10 medium (supplemented with 10% (v/v) FBS (Wisent, Montreal, Canada), 10 mM HEPES (Wisent, Montreal, Canada), Penicillin-streptomycin final concentration 100 μg/ml and L-glutamine final concentration 2 mM (life technologies, Burlington, ON). Cells were maintained at 37 °C in 5% CO_2_ incubator.

### Genomic DNA extraction

Approximately 1 × 10^6^ cells were collected and DNA was extracted with Purelink Genomic DNA mini kit (Invitrogen, Carlsbad, CA, USA), according to the manufacturer’s instructions. gDNA was then submitted to PCR reaction for further sequencing analysis. PCR conditions were implemented according to the manufacturer’s instruction with Phusion high-fidelity DNA polymerase (NEB, Ipswich, MA, USA). For all primers temperatures and cycles conditions are: 2 min at 98 °C followed by 40 cycles of 98 °C for 10 s, 64 °C for 30 s and 72 °C for 30 s, followed by a final extension step at 72 °C for 5 min. (See Supplementary Data Table 4 for primer sequences).

### RNA isolation and RT-qPCR

Total cellular RNA was isolated from DE0193, and DE0194 cells using TRIzol reagent (Life Technologies) according to the manufacturer’s instructions. cDNA was prepared from 1 μg of total RNA in 20 μl reaction volume using the RT omniscript (Applied Biological Material) according to the manufacturer’s instructions and diluted in 1/5. BrightGreen real-time PCR was performed with LightCycler system (Roche technology) and BrightGreen Express 2x qPCR MasterMix (Applied Biological Material) to amplify Human TTLL11 transcript 1 (NM_001139442) and transcript 2 (NM_194252). Amplification mixture (20 μl) contained 5 $\upmu$l of template diluted cDNA, 2× Mix and 300 nM forward and reverse primers. The cycle conditions were set as follows: 95 °C for 30 s, (95 °C for 5 s, 60 °C for 30 s) ×40 cycles, melting curve and cooling according to specific guidelines for LightCycler. All reactions were run in triplicate for each testing primer couple and GAPDH was run in duplicate to be used as the normalizing gene. The 2^−ΔΔCt^ method was used to calculate relative mRNA levels. (See supplemental Table 4 for primer sequences).

### Protein extraction and Western blot

Cell protein lysate were obtained from DE0194 and DE0193 fibroblasts using RIPA buffer (Pierce, Thermo-Fisher Scientific) (25 mM TrisHCl pH 7.6, 150 mM NaCl, 1% NP-40, 1% sodium deoxycholate, 0.1% SDS), containing protease and phosphatase inhibitors (Roche diagnostic). Resolved proteins were transferred to nitrocellulose membrane (Millipore) and then probed with different primary antibodies: TTLL11 (#PA-46070, Thermo-Fisher Scientific 1/500), GT335 (Adipogen #AG-20B-0020 1/1000), PolyE (Adipogen #IN105 1/1000) and $\upbeta$-actin (Santa Cruz Biotechnology #sc-47778). Membranes were then incubated with horseradish peroxidase-conjugated secondary antibody for chemiluminescent substrate visualization (ECL Plus, Amersham Biosciences).

### Immunofluorescence

Cells were cultured in an eight-well-chamber glass slide (Fisher scientific cat #354108). Mutant and WT cells were treated with medium without FBS for 24h before processing to immunofluorescence. Cells were fixed with (70% ethanol/0.2% triton on ice) and permeabilizated with 0.1% triton in PBS (PBST) then incubated with GT335 (Adipogen #AG-20B-0020 1/1000), PolyE (Adipogen #IN105 1/1000), anti-TTLL11 (thermofisher cat #PA5-46070 1/250) and anti-acetylated-$\upalpha$-tubulin (mouse monoclonal antibody, Sigma Aldrich cat #T7451 1/2000) antibodies diluted in 2% BSA/PBST. They were incubated for 1 h at room temperature (RT). Cells were then washed three times with PBST and incubated with secondary antibodies Alexa fluor 488 anti-rabbit (life technologies cat #A11008 1/500) and Alexa fluor 555 anti-mouse (Life technologies cat #A21422 1/500) for 1 h at RT. Mounting was done using prolonged gold anti-fade reagent with diamidino-2-phenylindole dye for fixed cells (DAPI) (Life technologies cat #P36931). Images were taken using Leica microscopy.

### Zebrafish maintenance

Adults, larvae and embryos were raised at 28.5 °C on a 14 h:10 h light:dark cycle. Adults and embryos were maintained using standard methods provided by the zebrafish book^92^. We used 0.02% tricaine (MS-222; Sigma Chemical, St. Louis, MO) in clean tank water to anaesthetize fish prior to all procedures.

### *Ttll11* knockdown in zebrafish

A translation blocking antisense morpholino oligonucleotide (MO) targeting the *ttll11* gene was designed and synthesized by Gene Tools LLC (USA). The sequence of the *ttll11*-MO was 5′-GGCTGATTTGTTATCTCATCTAGGT-3′, while that of the control-MO, was 5′- CCTCTTACCTCAGTTACAATTTATA-3′. MOs were diluted to 1 ng/nl, and approximately 3nl was injected into fertilised zebrafish eggs at the one- to two-cell stage using a Picospritzer III microinjector. Embryos were stage matched, anaesthetised using 0.02% MS222 in E3 embryo medium and observed under a stereomicroscope (SZX16 Olympus).

### *Ttll11 * mutant zebrafish

We used the CRISPRscan software^93^ to design the sgRNA to target the sequence **GAG**GTAGATGCCATCCCCTT**GGG** (with **PAM**, Protospacer Adjacent Motif). The designed sgRNA, taatacgactcactataGGGGTAGATGCCATCCCCTTgttttagagctagaa has no identified off-targets using CRISPR-scan. All injections were performed in 1-cell stage embryos from WT strain. Each embryo was injected with around 1.7 nl solution containing 30 ng/$\upmu$l of sgRNA and 100 ng/$\upmu$l of Cas9 mRNA. Mutants were selected by using HRM (High-resolution melting) as described previously^94^. To date, the transgenic lines have been outcrossed for more than 4 generations to dilute out potential off target mutations.

### Three-dimensional imaging and reconstruction

Three months old WT and *ttll11* mutant zebrafish were collected and fixed in 4% paraformaldehyde (PFA) overnight at 4 °C and three-dimensional Imaging and Reconstruction of Zebrafish spine underwent a micro-CT scan (SkyScan 1072 High Resolution Desktop Micro-CT System, Microtomograph, SkyScan) for three-dimensional (3D) visualization of the skeleton after 3D imaging and subsequent reconstruction. Acquisition parameters for the scan were as follows: 35 kV, 215 $\upmu$A, step rotation of 0.9°, pixel size 4-7 microns; images were reconstructed using NRecon (Version: 1.6.1.3).

The general body shape of 8 days-old larvae was also analysed under a stereomicroscope (Leica M205 FA).

### Retinal histology and immunostaining

Adult zebrafish were decalcified after micro-CT examination and embedded in paraffin wax prior to transverse sectioning. Eye tissue sections (6 μm) were then mounted on microscope slides and stained with hematoxylin eosin (H&E) following standard protocol. For immunostaining, eye tissue sections were deparaffinized in xylene, rehydrated in a graded series of ethanol, washed 3 times in PBS, and permeabilized for 30 min in 4% Triton X-100 containing 10% goat serum and 2% bovine serum albumin (BSA). Then retinal sections were incubated with the zpr1, zpr3 antibodies obtained from the Zebrafish International Resource Center^95^ or 3A10 antibody obtained from Hybridomas Bank (cat# AB_531874) (1/500) and acetylated-α-tubulin (Sigma Aldrich cat #T7451) (1/2000) simultaneously during 24 h at 4 °C. Tissue sections were washed several times in PBS and then incubated with the secondary antibody conjugated with Alexa Fluor 488 (life technologies cat# A11008 1/500) and Alexa Flour 555 (Life technologies cat # A21422 1/500) for 1 h at RT. Images were acquired using confocal microscope.

### Statistical analysis

All data values are given as means ± SD. Statistical analyses were performed, and data were plotted in SigmaPlot 11.0 (Systat Software Inc., San Jose, CA, USA). One-way ANOVAs and Fisher LSD tests were used to determine significance of normally distributed and equal variance data. Kruskal–Wallis ANOVA and Dunn’s method of comparison were used for non-normal distributions.

### Study approval

All animal procedures applied in this study were carried out in accordance with the guidelines set out by the Canadian Council for Animal Care (CCAC), the CHU Sainte-Justine Research Center, and the Comité de Déontologie de l’Expérimentation sur les Animaux (CDEA), which is the local animal care committee at the University of Montreal, Canada. The study was carried out in compliance with the ARRIVE guidelines (Animal Research: Reporting of *In Vivo* Experiments). This study was approved by the ethics committee for CHU Sainte-Justine Research Center, University of Montreal (ZF-09-60/Category B).

All procedures involving humans were carried out in accordance with the guidelines set out by the ethics committee of CHU Sainte-Justine Research Center. Written informed consent was received from participants and from legally authorised representative of minor participants prior to enrollment.

### Web resources

- VarAFT: https://varaft.eu/.
- UMD-predictor: http://umd-predictor.eu/_.
- HSF: http://www.umd.be/HSF3/.
- Mutation Taster: http://www.mutationtaster.org/.
- Ion Reporter: https://ionreporter.thermofisher.com/ir/.
- OMIM: https://www.omim.org/.
- Ensembl Genome Browser: http://useast.ensembl.org/index.html.
- UniProt: https://www.uniprot.org/.

## Supplementary Information

Supplementary Information 1.

Supplementary Information 2.

Supplementary Information 3.

Supplementary Information 4.
