# Supplementary material for: Genetic variant of TTLL11 gene and subsequent ciliary defects are associated with idiopathic scoliosis in a 5-generation UK family
Source: Sci Rep. 2021 May 26;11:11026. doi: 10.1038/s41598-021-90155-0 (PMC8155187; doi:10.1038/s41598-021-90155-0)
Supplement: Supplementary file 1 — Supplementary Information 1. [file 41598_2021_90155_MOESM1_ESM.pdf]

# Genetic variant of *TTLL11* gene and subsequent ciliary defects are associated with idiopathic scoliosis in a 5-generation UK family.

Hélène Mathieu[1], Shunmoogum A Patten[2], Jose Antonio Aragon-Martin[4], Louise Ocaka[3], Michael Simpson[6], Anne Child[5] *, Florina Moldovan[1, 7] *. (co – corresponding authors)

[1]CHU Sainte-Justine Research Center, Montréal, Québec, H3T1C5, Canada.

[2]INRS- Centre Armand-Frappier Santé et Biotechnologie, Laval, Québec, H7V1B7, Canada.

[3]Centre for Translational Omics – GOSgene, Department of Genetics and Genomic Medicine, UCL GOSH Institute of Child Health, 30 Guilford Street, London, WC1N 1EH, United Kingdom.

[4]NHLI, Imperial College, Guy Scadding Building, London, SW3 6LY, United Kingdom.

[5]Marfan Trust, NHLI, Imperial College, Guy Scadding Building, London, SW3 6LY, United Kingdom.

[6]Genetics and Molecular Medicine, King's College London, SE1 1UL, United Kingdom.

[7]Faculty of Dentistry, Université de Montréal, Montréal, Québec, H3T 1J4, Canada

*****Correspondence: Florina Moldovan, CRCHU Sainte Justine, 3175 Côte Sainte-Catherine, 2.17.026, Montréal (Québec) H3T 1C5, Canada; +15143454931, extension 5746; florina.moldovan@umontreal.ca.

## Supplementary Data

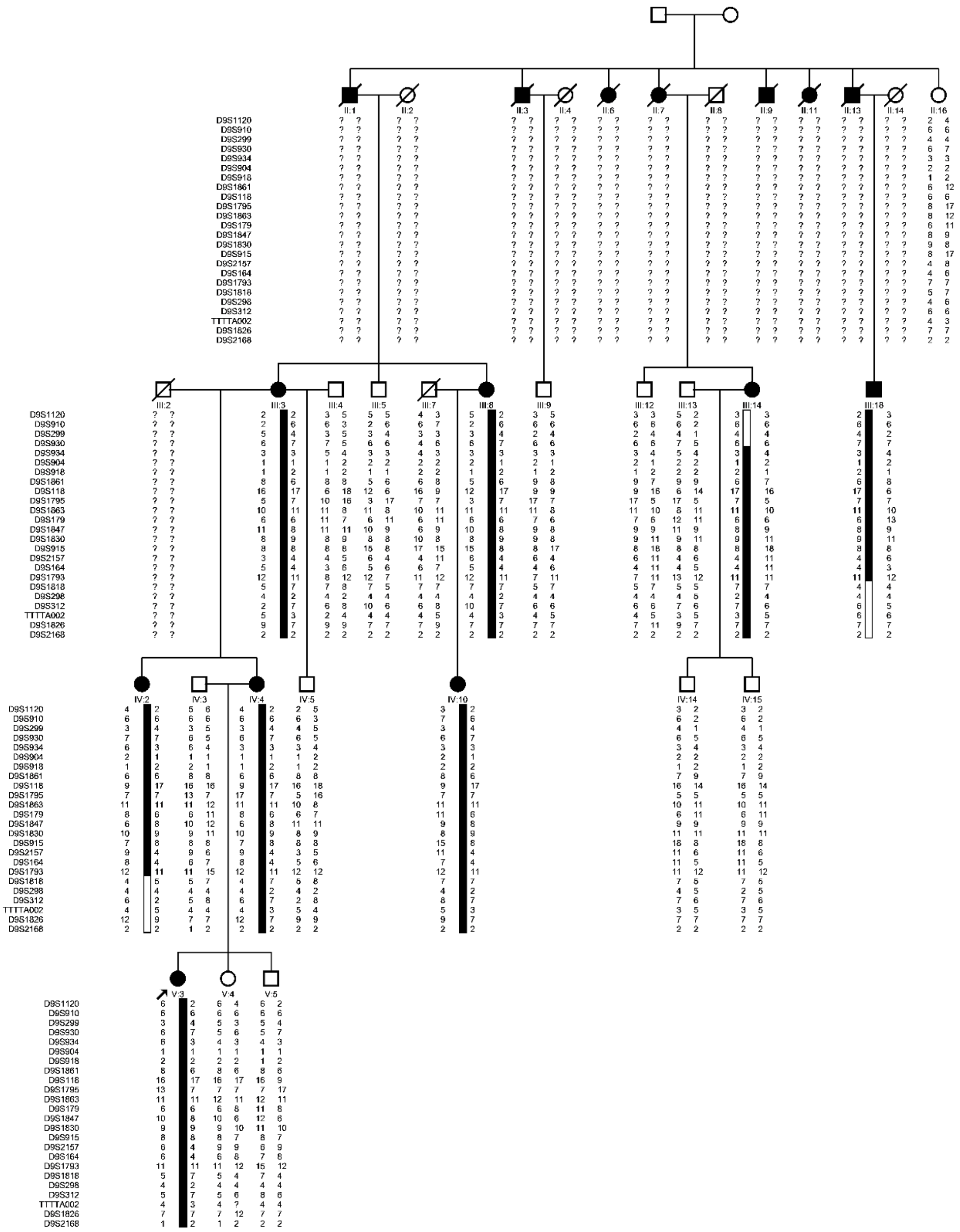

**Figure 1: Cosegregation of the 9q31.2-q34.2 locus with AIS in SC32 family.** Linkage analysis identify the 9q31.2-q34.2 locus as a candidate region for AIS as it perfectly cosegregate with the disease for the entire 5-generation family. Open circles and squares indicate unaffected individuals. Blackened circles and squares indicate affected females and males, respectively. The arrows indicate family proband.

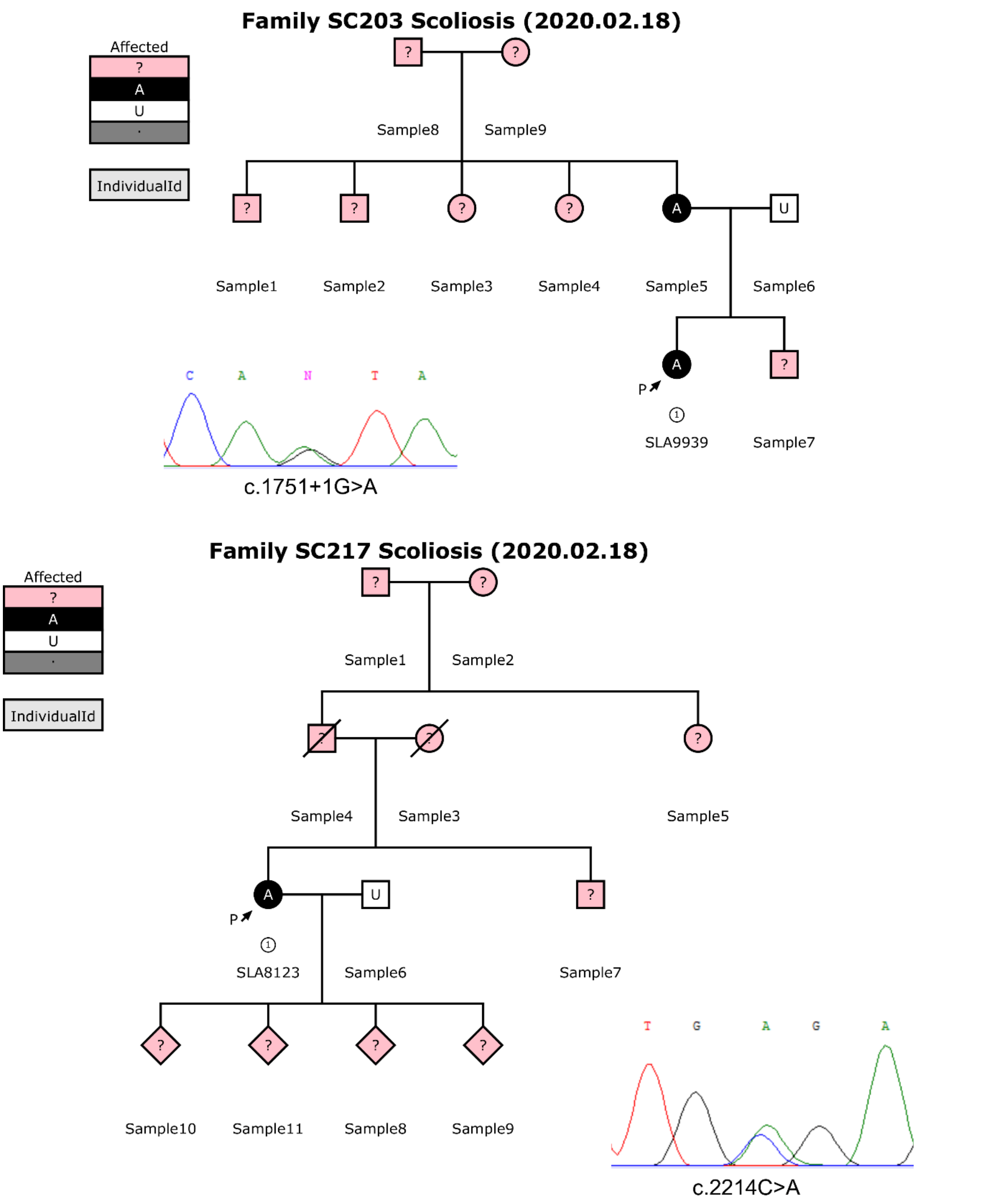


**Figure 2: Pedigree of UK Family SC203 and SC217 in which putative gene mutations has been found.** Open circles and squares indicate unaffected individuals. Blackened circles and squares indicate affected females and males respectively. The arrows indicate family proband. Chromatograms show the sequence obtained by Sanger sequencing of gDNA from both proband.

| Variant GRCh37 Pos | rs ID | Alleles | EA Allele # | EA Genotype # | MAF (%) | Avg. Sample Read Depth | Genes | mRNA Accession # | GVS Function | cDNA Change | Protein Change | Conservation (GERP) | PolyPhen2 (Class:Score) |
|---|---|---|---|---|---|---|---|---|---|---|---|---|---|
| 9:124584920 | unknown | R>A1 | A1=4/R=4648 | A1A1=1/A1R=2/RR=2323 | 0.086 | 6 | TTLL11 | NM_001139442.1 | frameshift | c.2348_2349insC | p.(P784Tfs*8) | -9.97 | unknown |
| 9:124751407 | rs148276492 | G>A | A=1/G=8599 | AA=0/AG=1/GG=4299 | 0.0116 | 54 | TTLL11 | NM_194252.2 | missense | c.1606C>T | p.(R536C) | 0.08 | benign:0.0 |
| 9:124751421 | rs142846947 | C>T | T=1/C=8599 | TT=0/TC=1/CC=4299 | 0.0116 | 60 | TTLL11 | NM_194252.2 | missense | c.1592G>A | p.(R531K) | -0.14 | benign:0.0 |
| 9:124751443 | unknown | R>A1 | A1=8/R=8246 | A1A1=0/A1R=8/RR=4119 | 0.0969 | 72 | TTLL11 | NM_194252.2 | frameshift | c.1569_1570insTT | p.(P524Ffs*4) | -2.22 | unknown |
| 9:124751578 | rs182367951 | T>C | C=1/T=8599 | CC=0/CT=1/TT=4299 | 0.0116 | 138 | TTLL11 | NM_001139442.1 | missense | c.1435A>G | p.(I479V) | 5.74 | probably-damaging:0.986 |
| 9:124751632 | rs367679783 | T>C | C=1/T=8599 | CC=0/CT=1/TT=4299 | 0.0116 | 104 | TTLL11 | NM_001139442.1 | missense | c.1381A>G | p.(I461V) | 3.42 | benign:0.083 |
| 9:124751637 | unknown | R>A1 | A1=1/R=8253 | A1A1=0/A1R=1/RR=4126 | 0.0121 | 99 | TTLL11 | NM_001139442.1 | frameshift | c.1375_1376insT | p.(S459Ffs*6) | 5.74 | unknown |
| 9:124751681 | rs149219458 | G>T | T=1/G=8599 | TT=0/TG=1/GG=4299 | 0.0116 | 92 | TTLL11 | NM_001139442.1 | missense | c.1332C>A | p.(F444L) | -2.65 | probably-damaging:0.997 |
| 9:124751733 | rs199708627 | C>T | T=1/C=8599 | TT=0/TC=1/CC=4299 | 0.0116 | 93 | TTLL11 | NM_001139442.1 | missense | c.1280G>A | p.(R427H) | 1.97 | benign:0.003 |
| 9:124751907 | rs145379039 | G>A | A=2/G=8598 | AA=0/AG=2/GG=4298 | 0.0233 | 84 | TTLL11 | NM_001139442.1 | missense | c.1106C>T | p.(A369V) | 5.12 | possibly-damaging:0.894 |
| 9:124751937 | rs375201152 | C>T | T=2/C=8598 | TT=0/TC=2/CC=4298 | 0.0233 | 99 | TTLL11 | NM_001139442.1 | missense | c.1076G>A | p.(R359H) | 5.12 | possibly-damaging:0.919 |
| 9:124751992 | unknown | R>A1 | A1=1/R=8253 | A1A1=0/A1R=1/RR=4126 | 0.0121 | 125 | TTLL11 | NM_001139442.1 | frameshift | c.1020del1 | p.(D341Mfs*57) | 4.89 | unknown |
| 9:124794020 | rs61740840 | G>T | T=66/G=8534 | TT=0/TG=66/GG=4234 | 0.7674 | 102 | TTLL11 | NM_001139442.1 | missense | c.945C>A | p.(F315L) | 4.6 | benign:0.01 |
| 9:124794038 | rs372169171 | C>G | G=1/C=8599 | GG=0/GC=1/CC=4299 | 0.0116 | 105 | TTLL11 | NM_001139442.1 | missense | c.927G>C | p.(W309C) | 5.51 | probably-damaging:0.999 |
| 9:124794099 | rs116884413 | C>T | T=32/C=8568 | TT=0/TC=32/CC=4268 | 0.3721 | 131 | TTLL11 | NM_001139442.1 | missense | c.866G>A | p.(R289K) | 5.51 | benign:0.291 |
| 9:124801631 | rs200141227 | C>T | T=1/C=8599 | TT=0/TC=1/CC=4299 | 0.0116 | 81 | TTLL11 | NM_001139442.1 | missense | c.749G>A | p.(R250H) | 6.07 | probably-damaging:0.999 |

**Table 1: List of the rare *TTLL11* SNPs reported by EVS database.** 16 SNPs, single-nucleotide substitutions or small indels, with minor allele frequency <1%, were reported by the Exome Sequencing Project (Exome Variant Server, NHLBI GO Exome Sequencing Project (ESP), Seattle, WA) that regroup more than 6000 exomes from different Sequencing projects.

A

| Chr | Start | End | Ref | Alt | Func refgene | Gene refgene | ExonicFunc refgene | AAChange refgene | 1000g 2015aug | CADD_phred | avsnp150 | MutationTaster _pred | UMD Score | UMD Prediction |
|---|---|---|---|---|---|---|---|---|---|---|---|---|---|---|
| 9 | 124584495 | 124584495 | G | A | UTR3 | TTLL11 | . | NM_001139442: c.*371C>T | 0.0626997 | . | rs7869045 | . | NA | NA |
| 9 | 124584560 | 124584560 | T | C | UTR3 | TTLL11 | . | NM_001139442: c.*306A>G | 0.0205671 | . | rs139112986 | . | NA | NA |
| 9 | 124585055 | 124585055 | G | T | exonic | TTLL11 | stopgain | NM_001139442: exon9:c.2214C>A: p.C738X | . | 38 | rs766983167 | D | 100 | Pathogenic |
| 9 | 124632735 | 124632735 | C | T | intronic | TTLL11 | . | . | 0.777955 | . | rs4384052 | . | NA | NA |
| 9 | 124736287 | 124736287 | C | T | splicing | TTLL11 | . | NM_001139442:exon 6: c.1751+1G>A | . | 23.5 | . | D | NA | NA |
| 9 | 124751086 | 124751086 | T | C | intronic | TTLL11 | . | . | 0.00698882 | . | rs143880966 | . | NA | NA |
| 9 | 124751110 | 124751110 | G | A | intronic | TTLL11 | . | . | 0.00159744 | . | rs72765951 | . | NA | NA |
| 9 | 124751312 | 124751312 | C | T | UTR3 | TTLL11 | . | NM_194252: c.*84G>A | 0.0151757 | . | rs55693486 | . | NA | NA |
| 9 | 124751361 | 124751361 | A | C | UTR3 | TTLL11 | . | NM_194252: c.*35T>G | 0.0830671 | . | rs41277110 | . | NA | NA |
| 9 | 124751390 | 124751390 | G | A | UTR3 | TTLL11 | . | NM_194252: c.*6C>T | 0.0672923 | . | rs41277112 | . | NA | NA |
| 9 | 124751443 | 124751443 | - | AA | exonic | TTLL11 | frameshift insertion | NM_194252:exon4: c.1569_1570insTT: p.P524fs | . | . | rs764409103 | . | NA | NA |
| 9 | 124794020 | 124794020 | G | T | exonic | TTLL11 | nonsynonymous SNV | NM_001139442; NM_194252:exon3: c.945C>A:p.F315L | 0.0305511 | 22.8 | rs61740840 | D | 21 | Polymorphism |
| 9 | 124855836 | 124855836 | G | A | UTR5 | TTLL11 | . | NM_001139442; NM_194252: c.-139C>T | . | . | rs554511731 | . | NA | NA |

B

| Chr | Start | End | Ref | Alt | Func.refgene | Gene.refgene | ExonicFunc. refgene | AAChange.refgene | 1000g2015aug_all | CADD_phred | avsnp150 | MutationTaster _pred | UMD Score | UMD Prediction |
|---|---|---|---|---|---|---|---|---|---|---|---|---|---|---|
| 9 | 124585055 | 124585055 | G | T | exonic | TTLL11 | stopgain | NM_001139442:exon 9: c.2214C>A:p.C738X | . | 38 | rs766983167 | D | 100 | Pathogenic |
| 9 | 124736287 | 124736287 | C | T | splicing | TTLL11 | . | NM_001139442:exon 6: c.1751+1G>A | . | 23.5 | . | D | NA | NA |
| 9 | 124751443 | 124751443 | - | AA | exonic | TTLL11 | frameshift insertion | NM_194252:exon4: c.1569_1570insTT: p.P524fs | . | . | rs764409103 | . | NA | NA |
| 9 | 124794020 | 124794020 | G | T | exonic | TTLL11 | nonsynonymous SNV | NM_001139442; NM_194252: c.-139C>T | 0.0305511 | 22.8 | rs61740840 | D | 21 | Polymorphism |

**Table 2: List of the SNPs identified by AmpliSeq in *TTLL11* gene from a French-Canadian and British AIS population.** A. 13 SNPs were reported prior any filtering. B. After filtering: MAF < 1%, single-nucleotide substitutions and small indels, 4 SNPs were selected.

| Subject | Cobb angle at diagnosis (°) | Type of curve | Treatment |
|---|---|---|---|
| III: 3 | 9/6/0 Scoliometer | Thoracic | Jacket for 6 months – 1 year<br>Back board up to 2 years |
| III: 8 | 6/7/5 Scoliometer | Thoracolumbar | Exercise |
| III: 14 | - | - | Back board from birth to 2 years old |
| III: 18 | - | - | None |
| IV: 2 | 7/7/4 Scoliometer | Thoracolumbar | Exercise and brace age 13 - 16 years old |
| IV: 4 | 6/10/4 Scoliometer | Thoracolumbar | None |
| IV:6 | 5/8/3 Scoliometer | Thoracolumbar | Exercise and physiotherapy |
| IV: 10 | 5/8/3 Scoliosis | - | Exercise and Physiotherapy |
| V: 3 Proband | 38 | Thoracolumbar | Brace 8 - 12 years, spinal fusion surgery age 13 years old |
| SC203 | NK | NK | None |
| SC217 | NK | NK | Surgery |

**Table 3: Clinical data of affected female members of IS patients from family SC32, patient SC203 and SC217 (Ocaka et al., 2008)**

Table showing clinical information for affected female members of family SC32 in three generations with an average of three members affected in each generation, patient SC203 and SC217. Higher Cobb angle value indicates a greater curve, and thus severity of AIS. Three measurements were taken using scoliometer, as Thoracic, Thoracolumbar and lumbar level in forward bending position.

| **qPCR primers for *TTLL11* detection** | |
|---|---|
| TTLL11-201-F (transcript variant 1 specific) | 5’ – GGACTCAGGGATGTGTCTGC – 3’ |
| TTLL11-201-R (transcript variant 1 specific) | 5’ – GGTGGTACTCGCAAAGGTCA – 3’ |
| TTLL11-t2-F (transcript variant 2 specific) | 5’ – TGCACCGCATCTTTATGCAC – 3’ |
| TTLL11-t2-R (transcript variant 2 specific) | 5’ – GCAATGGTTACCTGGAAGCAC – 3’ |
| GAPDH-FW | 5’ – ACCACAGTCCATGCCATCAC – 3’ |
| GAPDH-RW | 5’ – TCCACCACCCTGTTGCTGTA – 3’ |
| **Primers for PCR and sanger sequencing *TTLL11* exon 4** | |
| TTLLE4-FW1 | 5’ – CAACTTCATCCACTCGGACA – 3’ |
| TTLLE4-RV1 | 5’ – CACAGCACCACCAACACTG – 3’ |
| **Primers for HRM and sanger sequencing of zebrafish *ttll11* exon 4** | |
| HRM-ttll11-F | 5’ – CCTGCTCATCGATAAACTCAAG – 3’ |
| HRM-ttll11-R | 5’ – CCTGGACAAGCCTTCTTTAGC – 3’ |
| SEQ-ttll11-F | 5’ – CGGACAGTGGATCCCAAG – 3’ |
| SEQ-ttll11-R | 5’ – CTGCAGCTGTCAGAGTGGAC – 3’ |

**Table 4: qPCR primers and PCR primers for TTLL11 mRNA detection and sequencing analysis respectively.**

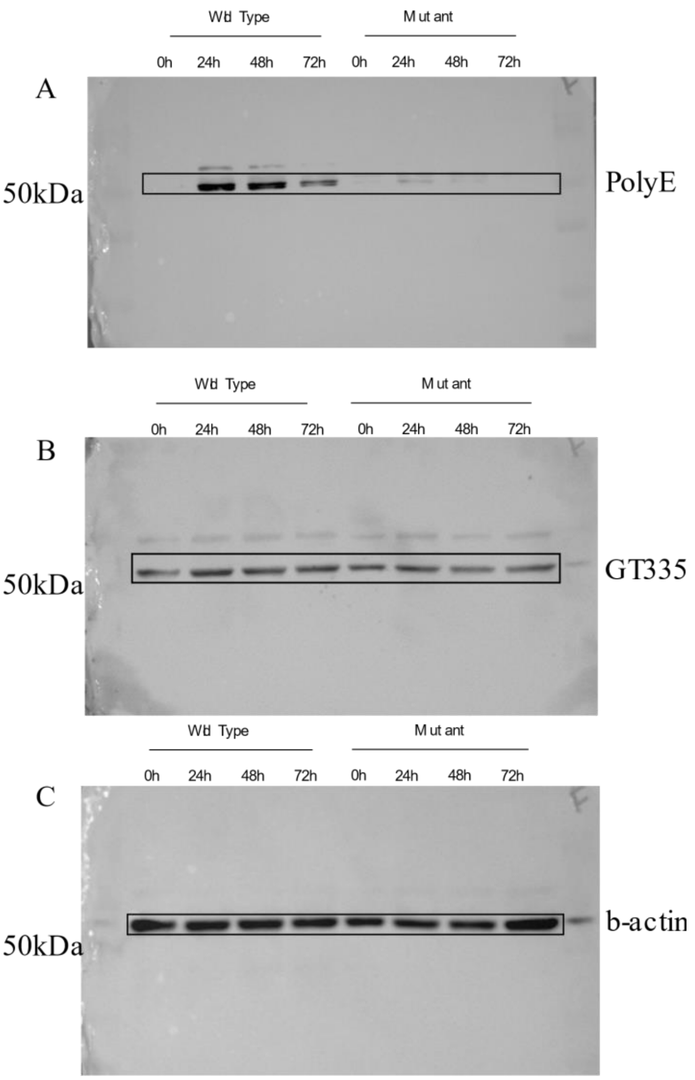


Figure 3: Protein polyglutamylation of Wild type (DE0194) and mutant (DE0193) fibroblasts treated with medium without FBS for 0, 24, 48 and 72 hours analysed by western blot (GT335 (B) and PolyE (A) antibodies) reveal the loss of long polyglutamylate chains for the mutant DE0193 cells. (full blots)
